# Correction to “lncRNA XLOC013218 Promotes Cell Proliferation and TMZ Resistance by Targeting the PIK3R2‐Mediated PI3K/AKT Pathway in Glioma”

**DOI:** 10.1111/cas.70131

**Published:** 2025-06-23

Zhou J, Xu N, Liu B, et al. lncRNA XLOC013218 promotes cell proliferation and TMZ resistance by targeting the PIK3R2‐mediated PI3K/AKT pathway in glioma. *Cancer Sci*. 2022; 113:2681–2692. doi:10.1111/cas.15387

In “3 | RESULTS” section, the text “3.4 | PIK3R2 knockdown reverses the enhanced TMZ sensitivity and promotes the cell survivability effect of XLOC” was incorrect.

This should have read: “3.4 | PIK3R2 knockdown partly reverses XLOC‐mediated TMZ resistance in GBM cells”.

In “3.4 | PIK3R2 knockdown reverses the enhanced TMZ sensitivity and promotes the cell survivability effect of XLOC” section, the text “To further explore whether PIK3R2 is involved in the effect of XLOC or not, sh‐PIK3R2 were transfected into cells stably overexpressing XLOC (U87 and U251 V‐XLOC) (Figure 4A). Knockdown of PIK3R2 significantly decreased the sensitivity of GBM cells to TMZ with lower IC50 values (Figure 4B) and suppressed cell viability with TMZ (50μg/ml) treatment when compared with the control groups (Figure 4C, D).” was incorrect.

This should have read: “To further explore whether PIK3R2 is involved in the effect of XLOC, sh‐PIK3R2 was transfected into cells stably overexpressing XLOC (U87 and U251 V‐XLOC) (Figure 4A). Knockdown of PIK3R2 significantly increased the sensitivity of GBM cells to TMZ, as indicated by lower IC50 values (Figure 4B), and reduced cell viability upon TMZ (50μg/ml) treatment compared to the control groups (Figure 4C, D).”

In “3.7 | XLOC as a candidate therapeutic target” section, the text “Immunohistochemistry analysis revealed that PIK3R2 and Ki67 expression was weakly detected, whereas increased C‐Cas3 expression was observed in XLOC‐overexpressed tumor tissues.” was incorrect.

This should have read: “Immunohistochemistry analysis revealed that PIK3R2 and Ki67 expression was increased, whereas C‐Cas3 expression was suppressed in XLOC‐overexpressed tumor tissues.”

In “3.3 | PIK3R2 is the potential target of XLOC” section, the text “To determine the subcellular localization of XLOC, RNA in situ hybridization (RNA‐ISH) was conducted” is incorrect.

This should have read: “FISH was conducted to determine the subcellular localization of XLOC”.

In “Figure 1” caption, the text “*p < 0.05, **p < 0.01, ***p < 0.001” is incorrect.

This should have read: “*p < 0.05, ***p < 0.001”.

In “Figure 4(D)”, the image is incorrect (The Y‐axis “2.5” is redundant).

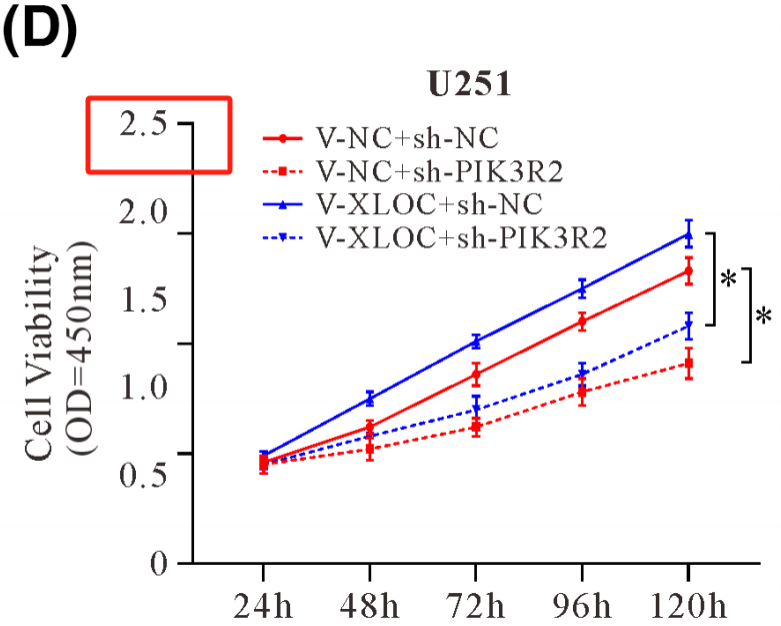

The correct Figure 4(D) should be:

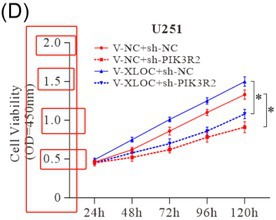

We apologize for these errors.

